# Optimization of Therapy in Patients with Epilepsy and Psychiatric Comorbidities: Key Points

**DOI:** 10.2174/1570159X20666220526144314

**Published:** 2023-06-15

**Authors:** Francesco Pisani, Laura Rosa Pisani, Maria Antonietta Barbieri, Jose de Leon, Edoardo Spina

**Affiliations:** 1 Department of Clinical and Experimental Medicine, University of Messina, Italy;; 2 Cutroni-Zodda Hospital Neurology Unit, Barcelona PG, Messina, Italy;; 3 Mental Health Research Center at Eastern State Hospital, Lexington, KY, USA and Psychiatry and Biomedical Research Centre in Mental Health Net (CIBERSAM), Santiago Apostol Hospital, University of the Basque Country, Vitoria, Spain

**Keywords:** Epilepsy, psychiatric disorders, antiseizure medications, drug interactions, seizure threshold, adherence

## Abstract

Psychiatric disorder comorbidity in patients with epilepsy (PWE) is very frequent with a mean percentage prevalence of up to 50% and even higher. Such a high frequency suggests that epilepsy and psychiatric disorders might share common pathological pathways. Various aspects contribute in making the matter very complex from a therapeutic point of view. Some antiseizure medications (ASMs), namely valproic acid, carbamazepine, and lamotrigine, have mood-stabilising effects and are routinely used for the treatment of bipolar disorder in patients who do not have epilepsy. Pregabalin and, to a lesser extent, gabapentin, exerts anxiolytic effects. However, several ASMs, in particular levetiracetam, topiramate, and perampanel, may contribute to psychiatric disorders, including depression, aggressive behaviour, and even psychosis. If these ASMs are prescribed, the patient should be monitored closely. A careful selection should be made also with psychotropic drugs. Although most of these can be safely used at therapeutic doses, bupropion, some tricyclic antidepressants, maprotiline, and clozapine may alter seizure threshold and facilitate epileptic seizures. Interactions between ASMs and psychotropic medication may make it difficult to predict individual response. Pharmacokinetic interactions can be assessed with drug monitoring and are consequently much better documented than pharmacodynamic interactions. Another aspect that needs a careful evaluation is patient adherence to treatment. Prevalence of non-adherence in PWE and psychiatric comorbidities is reported to reach values even higher than 70%. A careful evaluation of all these aspects contributes in optimizing therapy with a positive impact on seizure control, psychiatric wellbeing, and quality of life.

## 1 INTRODUCTION

Psychiatric disorders, especially anxiety and depression, are frequently observed in patients with epilepsy (PWE), and prevalence percentages of up to 50% and even higher have been reported [1-4]. In various investigations the highest values have been found in patients with specific forms of epilepsy, namely refractory and/or temporal lobe epilepsy (TLE) [3, 5, 6]. A frequent association between epilepsy and psychiatric disorders has been documented also in paediatric age [7, 8]. Apart from psychosocial factors and frequent stigma [9], a shared pathogenetic mechanism between the two conditions probably underlies the frequent association between epilepsy and psychiatric disorder [10-12]. On this basis, it might be very helpful for the clinician to have an in-depth knowledge of all the various related aspects, including the overall clinical condition of that patient and the complete pharmacological profile of the drugs chosen. There is no firm evidence that some antiseizure medications (ASMs) may induce psychotic disorders or depressive effects also associated with suicidal thought and behaviour [13-15]; on the other hand, some antidepressant or antipsychotic drugs may decrease seizure threshold [16-20]. Another key point that has to be taken into account when treating PWE and comorbid psychiatric disorders is the possibility of clinically relevant drug interactions (DIs) between ASMs and several antidepressants, antipsychotics, and anxiolytics.

An additional aspect that should not be underestimated in PWE and psychiatric disorders is drug adherence. This is known to be a relevant problem in both pathologies and a frequent cause of therapeutic failure [21-24]. Treatment adherence can be also hindered by memory disturbances and cognitive impairment induced by both ASMs and psychotropic drugs [25, 26].

The present article highlights selected topics that are relevant to optimize therapy in PWE and psychiatric comorbidities.

## 2 EPIDEMIOLOGY OF PSYCHIATRIC DISORDERS IN PATIENTS WITH EPILEPSY

Available literature data indicate that psychiatric disorders occur in a higher percentage in PWE than in the general population with an increased risk of 2 to 5-fold [1, 4, 27-29]. One out of three PWE has a diagnosis of a psychiatric disorder over lifetime [4]. The risk is higher in patients affected by specific forms of epilepsy, and prevalence values of 52% and 54% have been reported in patients with drug-resistant epilepsy and TLE, respectively [3, 6, 30]. Considering the type of epilepsy, personality and anxiety disorders have been reported to be more frequent in patients with idiopathic generalized epilepsy (IGE), while depressive and psychotic disorders appear to be more frequent in those with focal-onset seizures and drug-resistant epilepsy [2, 31]. In particular, diagnosis of a psychiatric disorder has been reported in approximately 43-51% of patients affected by juvenile myoclonic epilepsy [32, 33], 43.1% of patients with TLE and mesial temporal sclerosis [31] and 33% of patients with mesial TLE secondary to hippocampal sclerosis [33]. Concerning specific psychiatric disorders, data from recent literature show differing results. In a systematic review, mood/ affective disorders, including major depression, showed the highest prevalence values, up to 23%, followed by anxiety disorders (up to 15.6%), personality disorders and psychotic disorders (up to 4-11%) [2]. In another review, anxiety was more prevalent than depressive symptoms (44.8-52.9% *vs*. 30.8-49.9%) [29]. High prevalence values have been also reported for sleep disturbances, especially insomnia (61.4%) followed by excessive daytime sleepiness (EDS) and restless leg syndrome (RLS) (35.7% and 28.6%, respectively) [34].

Concerning gender, depression has been observed to occur more frequently in females than in males with epilepsy [29, 35]. According to a relatively small study based on self-report questionnaires [36], the incidence of comorbid anxiety and depression in PWE was similar for men and women, but predictors for anxiety and depression differed between genders: male patients were more likely to be affected by psychosocial factors, while female patients were more influenced by epilepsy itself.

Psychiatric disorders negatively influence cognitive and social functioning in PWE and are likely to decrease their quality of life (QOL) substantially, regardless of the seizure control itself [29, 37, 38]. Coexistence of psychiatric disorders has been associated with a poor response to ASMs and increased morbidity and mortality [39]. Some of the factors that might contribute to this poor response include lack of facilities for diagnosing psychiatric disorders, lack of appropriate treatment protocols either with medication or with psychological support [27, 28, 40]. Sudden unexpected death occurs in approximately 25% of PWE and psychiatric comorbidities regardless of the diagnostic category of the latter [39].

## 3 PSYCHOTROPIC EFFECTS OF ANTISEIZURE MEDICATIONS

ASMs can display beneficial or adverse psychotropic effects on mood, behaviour or cognition and knowledge of these properties is essential for a correct selection and optimization of therapy in PWE and psychiatric comorbidities [41-44]. Concerning the mechanism underlying these psychotropic effects, ASMs have different pharmacological actions which are likely to be responsible for the antiseizure activity but also for their psychiatric and behavioural effects. With regard to this, the molecular targets of ASMs for their psychotropic properties have been extensively discussed [45, 46] and recently updated [47].

### 3.1 Positive Effects

Carbamazepine, valproic acid, and lamotrigine, have mood-stabilising properties and are routinely used for the treatment of bipolar disorder in non-epileptic patients [48-50]. Carbamazepine and valproic acid are both highly effective in the treatment of acute mania while lamotrigine is more effective for preventing recurrent depressive episodes [51-53]. Lamotrigine is indicated for maintenance treatment of bipolar disorder and, in particular, it was approved to be used in patients with bipolar disorder type I [54]. Recent data support lamotrigine efficacy in acute bipolar depression [55]. However, it must be emphasized that the necessity to introduce lamotrigine slowly to decrease the probability of skin rash may limit the practical use of lamotrigine in an acute disorder. Other ASMs, including gabapentin, topiramate, oxcarbazepine, levetiracetam, tiagabine, or zonisamide, have been investigated for their potential mood-stabilising properties but results are still inconclusive [48] and they are not recommended as first-lines in guidelines for the treatment of bipolar disorder [56].

Gabapentin and pregabalin display anxiolytic effects and the latter has been approved in Europe for the management of generalized anxiety disorder [57-59].

### 3.2 Negative Effects

Various ASMs are known to cause psychiatric adverse effects (AEs) including depression, psychosis, behavioural changes (irritability, hyperactivity, agitation, and aggressive behaviour), and cognitive dysfunction [42, 60]. These negative effects are more common when ASMs are administered to PWE than when prescribed for non-epilepsy conditions [61]. A percentage of 15-20% of PWE has been reported to develop psychiatric AEs, the main risk factor being a personal history of psychiatric illness [62]. However, it is difficult to distinguish these drug-induced AEs from psychopathological manifestations of epilepsy itself or symptoms of actual psychiatric comorbidities. Psychiatric AEs may profoundly affect QOL and well-being and lead to suboptimal dosing and/or poor adherence to treatment [63].

#### 3.2.1 Depression and Risk of Suicide

Among ASMs, phenobarbital, primidone, topiramate, vigabatrin and to a lesser extent, felbamate, levetiracetam, tiagabine, and zonisamide, have been associated with the occurrence of symptoms of depression and close monitoring is recommended in patients with a history of depression [15, 43-46].

In 2008 a warning about an increased risk of suicide ideation and behaviour has been issued by the US Food and Drugs Administration (FDA) based on a meta-analysis of placebo-controlled clinical trials of 11 ASMs (carbamazepine, felbamate, gabapentin, lamotrigine, levetiracetam, oxcarbazepine, pregabalin, tiagabine, topiramate, valproate, and zonisamide) [64, 65]. The FDA meta-analysis documented that individuals treated with ASMs had an almost double, statistically significant increased risk of suicidal behaviour and ideation (4.3 per 1000) as compared to placebo patients (2.2 per 1000) [64, 65]. After this report, the FDA required that all manufacturers of drugs in this class include a warning in their labeling about the risk of suicidal thoughts or actions. Subsequent studies analyzed the FDA data and identified a number of methodological limitations [66-68]. In particular, while the FDA document indicated an increased risk of suicide with all ASMs, based on the available data, statistical significance was found only for topiramate and lamotrigine. Since then, many retrospective cohort and case-control studies investigated whether or not there is an association between ASMs and the risk of suicidal ideation and behavior, but contradictory results continue to be reported [69-72]. Arana *et al*. [71] examined the association between ASMs and suicide-related events (defined as suicidal attempts and completed suicides), using data collected as part of clinical practice in the United Kingdom. The use of antiepileptic drugs was not associated with an increased risk of suicide-related events among PWE (odds ratio [OR], 0.59; 95% confidence interval [CI], 0.35 to 0.98) or bipolar disorder (1.13; 0.35 to 3.61) but was significantly associated with an increased risk among patients with depression (1.65; 1.24 to 2.19) and among those with none of these conditions (2.57; 1.78 to 3.71).

A recent meta-analysis evaluated the risk of suicidality (ideation, attempts, and completed suicides) of 5 newer ASMs such as eslicarbazepine, perampanel, brivaracetam, cannabidiol, and cenobamate [73]. The analysis included 17 randomized clinical trials of these drugs, involving 5996 patients, of whom 4000 patients were treated with ASMs and 1996 with placebo. There was no evidence of increased risk of suicidal ideation (drugs *vs*. placebo overall risk ratio [RR], 0.75; 95% CI, 0.35 to 1.60) or attempt (RR, 0.75; 0.30 to 1.87) overall or for any individual drug.

Suicide in epilepsy is a complex phenomenon that is likely to be multifactorial, with biological, constitutional, and psychological variables implicated [61]. The overall risk of suicidal ideation or behaviour is about three times higher in PWE than in the general population [74]. A recent meta-analysis demonstrated an increased OR for the association between epilepsy and suicide attempts (pooled OR, 3.25; 95% CI, 2.69 to 3.92, *p* < 0.001) [75]. On this basis, firm conclusions cannot be drawn on the role of ASMs in suicidal thoughts and behaviour; patients must be carefully evaluated by physicians.

#### 3.2.2 Psychosis and Aggressive Behaviour

It has been reported that first-generation ASMs such as phenobarbital, benzodiazepines, and valproic acid are typically associated with hyperactivity and irritability in school- and preschool-aged children and individuals with intellectual disabilities [42, 60]. A number of ASMs, *i.e.*, ethosuximide, lamotrigine, levetiracetam, perampanel, phenytoin, tiagabine, topiramate, vigabatrin, and zonisamide, have occasionally been reported to cause psychosis [43]. Psychotic disorders are more commonly induced by second-generation ASMs, particularly lamotrigine, topiramate, and levetiracetam [13, 76]. However, the prevalence of psychosis was found to be relatively low, as 0.4% with lamotrigine [77], 3.7% with topiramate [78], and 1.2% with levetiracetam [79]. Other ASMs such as levetiracetam, topiramate, lamotrigine, and perampanel have been associated with the onset of aggressive behaviour in PWE [13, 80]. In a recent meta-analysis, the mean incidence for aggression was 2.5% for brivaracetam, 2.6% for levetiracetam, 4.4% for perampanel, and 0.5% for topiramate [81]. A 12-week, short-term, observational prospective study in patients with focal epilepsy, found no significant difference in terms of incidence and severity of psychiatric and behavioral side effects associated with perampanel in patients with TLE as compared to other focal epilepsies [82].

#### 3.2.3 Cognition

All ASMs can have a negative impact on cognitive performance with detrimental effects on attention, memory, executive function, and language [83, 84]. Available evidence indicates that lamotrigine, levetiracetam, and lacosamide are associated with less cognitive impairment as compared to topiramate, phenytoin, phenobarbital, and zonisamide [84, 85].

## 4 EFFECTS OF PSYCHOTROPIC DRUGS ON SEIZURE ACTIVATION

The risk of seizure occurrence induced by psychotropic drugs was recognized towards the middle of the last century with the introduction and the use of the antidepressant imipramine [86, 87] and antipsychotics [88]. Given the frequent association between psychiatric disorders and epilepsy, the matter has received great interest over the past years [89-93].

Available evidence indicates that some antidepressants are associated with a dose-dependent risk of seizures, as documented with maprotiline, clomipramine (usually at doses > 300 mg/day), amitriptyline (at doses > 200 mg/day), and bupropion immediate release formulation (at doses > 450 mg/day) [17, 94, 95]. The lower rate of seizures reported in clinical trials of slow release *vs*. immediate release bupropion formulations may be related to lower peak plasma concentrations with the slow release formulation, clearly suggesting that the proconvulsive effect of bupropion is dose- and concentration-dependent [96].

Some antipsychotics may also have proconvulsant effects [97]. Among first-generation antipsychotics, chlorpromazine appears to be the most epileptogenic with an increased risk of seizures at doses higher than 1000 mg/day which are rarely used in clinical practice [94]. Regarding second-generation antipsychotics, clozapine is associated with the highest, dose- and titration-dependent risk of seizures with prevalence rates of 1% at doses below 300 mg/day, 2.7% at doses between 300 and 600 mg/day, and 4.4% at doses above 600 mg/day [98, 99]. The risk of seizures was reported to increase with plasma clozapine levels greater than 600 μg/L or rapid upward titration [100]. Olanzapine and quetiapine, both chemically related to clozapine, may also cause epileptic seizures but the risk is much lower as compared to clozapine [99].

An analysis of seizure incidence in phase II and III clinical trials of psychotropic drugs approved by the FDA between 1985 and 2004 involving over 75000 patients confirmed an increased incidence of seizures for specific drugs: clozapine, olanzapine, quetiapine, among antipsychotics, clomipramine and bupropion, among antidepressants [101]. A subsequent extensive investigation based on the World Health Organization (WHO) adverse drug reactions (ADRs) database has indicated that, although a firm causal relationship was absent, the psychotropic drugs most frequently associated with seizure triggering were in descending order as follows: maprotiline, escitalopram, bupropion, clozapine, amoxapine, quetiapine, and trimipramine [102]. Following these two investigations, the risk of seizures with the use of psychotropic drugs has been examined in several reviews and research articles with uneven results. Selective serotonin reuptake inhibitors (SSRI), serotonin-noradrenaline reuptake inhibitors (SNRI), and first and second-generation antipsychotics have been reported to exert little or no effect on seizure frequency [103-105]. On the contrary, other studies have indicated that the risk of seizures is higher for all classes of antidepressants [106] or only for specific antidepressants, *i.e.*, imipramine, bupropion, and specific antipsychotics, *i.e.*, clozapine, olanzapine, and haloperidol [107]. A prospective, nationwide surveillance study was carried out in a large paediatric (< 18 years) population to identify drugs most commonly responsible for epileptic seizures in teenagers with new-onset seizures of unknown aetiology and who for various reasons, most frequently intentional overdose and medication error, ingested different drugs [108]. Among different classes of drugs ingested, antidepressants, specifically bupropion, but also citalopram and tricyclic antidepressants, were the most common medications responsible for overdose for paediatric drug-induced seizures [108]. Admittedly, no firm conclusions can be drawn from overdose data, about the risk of seizures with therapeutic doses of medication. More recently, no increment in seizure was observed with the use of psychotropic drugs in a population of PWE and intellectual disability [95], and in another study, the majority of psychotropic drugs, apart from bupropion and clozapine, were not pro-convulsant when used in therapeutic doses [20].

In experimental settings, similar results, namely that seizure threshold can be modified by single specific psychotropic drugs, have been documented. Fluoxetine and duloxetine, for example, reduced the development of spontaneous seizures in rat models of epilepsy when therapy began before the initiation of the seizures, but whereas duloxetine also reduced absence episodes, fluoxetine increased their occurrence [109]. In another study in genetically epilepsy-prone rats, the same authors have observed that, after regular drug administration, clozapine exerted the most marked pro-convulsant effects, followed by risperidone and olanzapine; quetiapine and haloperidol had only modest effects, and aripiprazole showed anticonvulsant properties [110]. Various mechanisms have been proposed to explain the seizure facilitation properties of psychotropic drugs, including their effects on GABAergic-glutamatergic balance, monoamines, and other putative neurotransmitters, G protein-coupled K+ channels, and other mechanisms [109-114]. However, even in the experimental context, available data are insufficient to identify the true mechanisms through which some psychotropic drugs are associated with an increased risk of seizures and which specific single drugs exhibit a consistent effect. Thus, based on available data, definitive conclusions cannot be drawn on their use in clinical practice. The most consistent findings suggest the following points: a) clozapine, bupropion, and possibly clomipramine are associated with an increased risk of epileptic seizures; b) the majority of psychotropic drugs used at low-therapeutic doses do not alter seizure threshold; c) SSRI and SNRI could exert also anti-seizure effects.

## 5 DRUG INTERACTIONS

Another clinically relevant aspect that has to be taken into account when treating PWE and comorbid psychiatric disorders is the possibility of DIs. In recent years, numerous comprehensive reviews have addressed this issue [94, 115-118]. DIs are typically classified into pharmacokinetic (PK) and pharmacodynamic (PD). Clinically significant PK DIs between ASMs and drugs for psychiatric disorders are described in detail in another article on this thematic issue.

### 5.1 PK

The majority of clinically significant PK DIs between ASMs and drugs used to treat comorbid psychiatric disorders occur at a metabolic level and usually involve the main drug-metabolizing enzymes, namely the cytochrome P450 (CYP) system and, to a lesser extent, the uridine diphosphate glucuronosyltransferase (UGT) system. Most compounds of these therapeutic classes undergo extensive hepatic biotransformation and share common metabolic pathways. Furthermore, a number of ASMs and psychiatric disorders drugs may influence the activity of drug-metabolizing enzymes acting as inducers or inhibitors. Based on the available knowledge of substrates, inhibitors, and inducers of different CYP and/or UGT isoforms, it is possible to anticipate and avoid potential DIs. However, it is necessary to point out that only a few theoretically predictable PK DIs between antiseizure and psychotropic drugs may have clinically relevant consequences. Indeed, the occurrence, magnitude, and clinical significance of these potential DIs strictly depend on a variety of drug-related (*e.g.*, potency and concentration/dose of the inhibitor/inducer, the therapeutic index of the substrate, the extent of metabolism of the substrate through the affected enzyme, presence of active metabolites), patient-related (*e.g.*, age, genetic predisposition), and environmental factors (*e.g.*, smoking, diet).

Some old ASMs, such as carbamazepine, phenytoin, phenobarbital, and primidone, are potent inducers of several drug-metabolizing enzymes and may significantly reduce the plasma concentrations and, possibly, the efficacy of some antidepressants (*i.e.*, sertraline, citalopram, mirtazapine, bupropion), and some antipsychotics (*i.e.*, risperidone, olanzapine, quetiapine, aripiprazole) [115, 119, 120]. Newer ASMs are generally associated with limited enzyme-inducing potential compared to first-generation compounds. However, oxcarbazepine (at dosages ≥ 1200 mg/day), and topiramate (at dosages ≥ 400 mg/day) can induce the activity of CYP3A4 and, possibly, some UGTs, whereas cenobamate, clobazam, eslicarbazepine, felbamate, and rufinamide are considered as mild inducers.

Among first-generation ASMs, valproic acid has traditionally been considered a broad-spectrum inhibitor of various drug-metabolizing enzymes including CYP2C9, CYP2C19, and CYP3A4, and some UGTs. However, recent evidence indicates that valproic acid may also be a dose-dependent mild inducer of some antipsychotics such as clozapine and olanzapine. Lamotrigine is a weak UGT inducer, while it does not appear to be a CYP inhibitor or inducer. Some of the newer ASMs may act as enzyme inhibitors. In this respect, cenobamate, eslicarbazepine, felbamate, oxcarbazepine, and topiramate are weak inhibitors of CYP2C19, while stiripentol is a potent inhibitor of CYP1A2, CYP2C19, CYP2D6, and CYP3A4.

Some newer antidepressants, such as fluoxetine, fluvoxamine, paroxetine, duloxetine, and bupropion, act as potent or moderate inhibitors of various CYPs and may therefore impair the elimination of ASMs metabolized *via* these isoforms. In this respect, it is well documented that fluoxetine and fluvoxamine may cause a clinically significant increase in plasma concentrations of phenytoin and valproic acid [121-125]. Conversely, most antipsychotics and benzodiazepines appear to be neither inhibitors nor inducers of drug-metabolizing enzyme systems and have not been reported to cause clinically relevant metabolically-based DIs with ASMs.

Clinicians should be aware of the potential risks of reduced efficacy when adding inducers or of increased ADRs when adding inhibitors, as well as the risks of AEs when discontinuing inducers or losing efficacy when discontinuing inhibitors. In some cases, in addition to a careful clinical evaluation, therapeutic drug monitoring of antiepileptic and/or psychotropic compounds may be a valuable tool to assess DIs and, therefore, to optimize drug treatment in PWE and associated psychiatric disorders.

### 5.2 PD

The PD DIs between ASMs and psychotropic drugs have not been systematically investigated. In general, PD DIs are not easy to study since they are not associated with any changes in plasma drug concentration. As a consequence, these interactions are more difficult to identify and measure than PK interactions. The limited available information on clinically relevant PD DIs between ASMs and the most important psychotropic medications (antidepressants, antipsychotics, benzodiazepines, and lithium) has recently been summarized [116].

PD DIs take place directly at the site of action of a drug or indirectly by interfering with another physiological mechanism. In that sense, the ASM modes of action involve different mechanisms including sodium and calcium channel blockade, potentiation of GABAergic transmission and inhibition of glutamatergic transmission; they likely interact at the PD level with psychotropic medications, which may act directly or indirectly on the same systems. These interactions are typically categorized as additive (*i.e.*, equal the sum of the effects of the individual drugs), synergistic (*i.e.*, combined effects are greater than expected from the sum of individual effects), or antagonistic (*i.e.*, combined effects are less than additive) and can be associated with beneficial or harmful effects.

Considering negative effects, the combination of ASMs with medication used to treat comorbid psychiatric disorders may occasionally generate decreased efficacy. As stated, some ASMs, such as phenobarbital, primidone, topiramate, vigabatrin, felbamate, levetiracetam, tiagabine, and zonisamide, are associated with an increased risk of depression. Although not fully characterized, it is reasonable to hypothesize that some ASMs might counteract the efficacy of antidepressant medications in PWE. However, some psychotropic medications may cause a dose-dependent decrease in seizure threshold and lead to seizure exacerbation.

More often, PD DIs are associated with an increased risk of ADRs. Thus, the concomitant administration of ASMs with other psychiatric medications may potentially worsen common ADRs, such as the increased risk of sedation, metabolic AEs including weight gain, hyperglycemia, and dyslipidemia, and sexual dysfunction [116]. Less frequent, but severe PD DIs may occasionally occur when combining antiseizure and psychotropic medications [116]. For example, the concomitant administration of ASMs and antidepressants may be associated with severe toxic effects, such as hyponatremia, arrhythmias, hepatotoxicity and bleeding [115]. Rare, but serious AEs including arrhythmias, pancreatitis, and hematological effects, may arise from ASM and antipsychotic combinations [118]. The ASM, carbamazepine, has the highest potential for causing neutropenia/agranulocytosis, and should not be combined with the antipsychotic, clozapine, due to a potentially increased risk of agranulocytosis [126, 127]. The co-administration of ASMs with lithium is generally accepted as safe and effective. However, concomitant treatment with valproate and lithium may generate additive AEs such as weight gain, sedation, gastrointestinal complaints, tremor, and cognitive impairment [126, 128]. Neurotoxin symptoms such as cerebellar signs (*i.e.*, ataxia and nystagmus), confusion, drowsiness, lethargy, and tremor have been described with the combination of carbamazepine with lithium, particularly in patients with pre-existing central nervous system disease [126, 128-131]. Notwithstanding, the pharmacological mechanisms underpinning these PD DIs require clarification.

Clinicians need to become more aware of the potential for dangerous combinations of the increasingly frequent co-prescription of ASM and psychotropic agents. It is not documented whether or not the combination may increase their risk, but pharmacological mechanisms suggest that possibility, and clinicians need to remember the potential for PD DIs in order to prevent or quickly diagnose them. A prior article summarized these PD DIs using a table format [116].

## 6 THE COMPLEX PROBLEM OF PATIENT NON-ADHERENCE TO TREATMENT

A partial or complete non-adherence of patients to prescribed treatment is a well-known cause of therapeutic ineffectiveness, especially in chronic diseases [132-134]. Non-adherence to treatment may be explained by several factors including negative beliefs about medications, fear of side effects, difficulty of the regimen, suboptimal physician-patient relationships, inadequate social support, and cost of treatment [132-134]. In PWE, non-adherence rates range from 30 to 60% [135, 136] and can result in poor seizure control, worsening of QOL, and increased risk of sudden death [136-138]. Similar results have been reported in populations of patients with psychiatric disorders. In particular, a recent systematic review has reported rates of noncompliance of 50% of patients with major depression, 56% of patients with schizophrenia, and 44% of patients with bipolar disorder [139]. Even higher values, *i.e.*, >70%, have been reported in other investigations in patients with specific diagnoses of schizophrenia [140] and major depression [141]. This matter can be complicated further because non-adherence to treatment can vary over time, and can be intentional (*e.g.,* fear of AEs) or unintentional (*e.g.*, missing medication by mistake or forgetting to take it) [142, 143].

Different multifactorial strategies have been proposed to improve medication adherence to treatment, including: improvement of the physician-patient relationship, use of long-acting formulations of medications to reduce the need for daily administration, patient education on medication benefits and risks resulting from therapy discontinuations, use of pictograms (pictures or symbols representing a word or phrase), use of special medication containers, and others [144-148]. If non-adherence is suspected, repeated therapeutic drug monitoring is extremely useful to explore this possibility [149]. In the case of low blood levels of a given medication, of course, a PK DI due to an enzyme-inducing drug should also be taken into consideration [150].

Based on the above-mentioned available data, non-compliance must be considered one of the primary causes of therapy failure, especially in PWE and psychiatric comorbidities [21, 151, 152].

## CONCLUSION

As outlined, psychiatric disorders coexist in approximately 30-50% of PWE, with a 2-5-fold increased risk of developing a psychiatric disorder when compared with the general population [1-4]. Such a high association frequency between pathologies suggests that common pathogenetic features are at play. In line with this evidence, the treatment of PWE and psychiatric comorbidities is highly complex (Table **1**).

Some ASMs are currently used as mood stabilizers (*e.g.*, carbamazepine, valproate, and lamotrigine) [17, 51] or anxiolytics (*e.g.*, pregabalin) [57]. However, several ASMs, in particular levetiracetam, topiramate, and perampanel, may contribute to psychiatric disorders, including depression occasionally associated with suicidal thought and behaviour, aggressive behaviour, and even psychosis [13-15]. Although most psychotropic drugs can be safely used at therapeutic doses, bupropion, some tricyclic antidepressants, maprotiline, and clozapine are associated with a dose-dependent risk of seizure induction [18, 20, 105, 107]. An important key point that has to be taken into account when treating PWE and comorbid psychiatric disorders is the possibility of clinically relevant DIs between ASMs and a number of antidepressants, antipsychotics, and anxiolytics. Another aspect that needs a careful evaluation is patient adherence to therapy. Given the nature of both pathologies, where behavioral and cognitive disturbances are associated, non-adherence is a frequent occurrence. In this respect, the prevalence of non-adherence in PWE and psychiatric comorbidities is reported to reach values even higher than 70% [21, 143, 151, 152].

To conclude, the treatment of PWE and psychiatric comorbidities is a complex task and requires a deep knowledge of the overall clinical condition of that patient and the pharmacological profile of the drugs chosen. Apart from the correct diagnosis of both pathology types and the aforementioned issues, many other aspects make treating these patients a challenge, including: the presence of psychogenic seizures, the presence of other diseases, epilepsy severity, psychiatric disorder severity, and genetic and hereditary factors. Therefore, therapeutic approaches, which consider all possible factors for a given patient, are crucially important for complex processes in personalized medicine for reducing the risk of treatment failure, AEs, and a worsening QOL [153].

## Figures and Tables

**Table 1:** Main key points in the treatment of PWE and psychiatric comorbidities.

**Key Point**	**Drugs**	**Effects**	**References**
Psychotropic effects of ASMs	Carbamazepine, valproic acid, lamotrigine Gabapentin, pregabalin Phenobarbital, primidone, felbamate, levetiracetam, tiagabine, topiramate, vigabatrin, zonisamide Lamotrigine, levetiracetam, perampanel, topiramate Phenobarbital, phenytoin, topiramate, zonisamide	Mood stabilisation Anxiolytic effect Depression Psychosis and/or aggressive behaviour Cognitive impairment	[48-50] [57-59] [43-46] [13, 76-80] [83-85]
Effect of psychotropic drugs on seizure activation	Bupropion, imipramine, maprotiline, clozapine SSRI, benzodiazepines	Lowering of seizure threshold Antiseizure effect	[94-96, 98, 99] [103]
PK DIs of clinical relevance	Phenobarbital, primidone, phenytoin, carbamazepine, oxcarbazepine (>1200 mg/day), topiramate (>400 mg/day) Fluoxetine, fluvoxamine	Lowering of blood levels of multiple psychiatric drugs including sertraline, citalopram, mirtazapine, bupropion / risperidone, olanzapine, quetiapine, and aripiprazole with the possible efficacy reduction Increase in blood levels of phenytoin and valproic acid with the possible occurrence of AEs	[115, 119, 120] [121-125]

## LIST OF ABBREVIATIONS

ADRs: Adverse Drug Reactions
AEs: Adverse Effects
ASMs: Antiseizure Medications
CI: Confidence Interval
CYP: Cytochrome P450
DIs: Drug Interactions
EDS: Excessive Daytime Sleepiness
FDA: Food and Drugs Administration
IGE: Idiopathic Generalized Epilepsy
OR: Odds Ratio
PD: Pharmacodynamic
PK: Pharmacokinetic
PWE: Patients with Epilepsy
QOL: Quality of Life
RLS: Restless Leg Syndrome
RR: Risk Ratio
SNRI: Serotonin-noradrenaline Reuptake Inhibitors
SSRI: Selective Serotonin Reuptake Inhibitors
TLE: Temporal Lobe Epilepsy
UGT: Uridine Diphosphate Glucuronosyltransferase
WHO: World Health Organization
